# Combined chest wall and liver hydatid cyst

**DOI:** 10.21542/gcsp.2023.32

**Published:** 2023-09-30

**Authors:** Feridoun Sabzi, Reza Faraji

**Affiliations:** 1 Department of General Surgery, School of Medicine, Kermanshah University of Medical Sciences, Kermanshah, Iran; 2 Tuberculosis and Lung Diseases Research Center, Ilam University of Medical Sciences, Ilam, Iran

## Abstract

Background: Hydatid cysts (HC) are primarily found in the liver, with secondary occurrences in the lungs and other organs. The presence of HCs in the anterior chest wall is notably rare, and even more so when associated with HCs in the liver.

Case Presentation: A 53-year-old male reported to our facility with a non-painful lump on his chest’s front wall. A thoraco-abdominal CT scan identified cysts within the chest wall’s subcutaneous layer, showing no spread to nearby soft tissues or involvement of the lungs and ribs. Despite an echinococcal test returning negative, the initial diagnosis leaned towards a dermoid cyst. After surgical removal and detailed examination, the cysts were confirmed as HCs. Further investigation revealed an additional liver HC. The patient was referred for surgery where he underwent laparotomy and drainage of cyst content.

Conclusion: This case underscores the importance of considering HCs when diagnosing palpable lesions on the chest wall, particularly in regions where HCs are endemic.

## Introduction

Echinococcal infestation is caused by the cestode stage of *Echinococcus* tapeworms of the family Taeniidae. The primary hosts are carnivores, whereas the intermediate hosts are livestock such as cattle and sheep and goats. Humans are infected by ingesting ova from vegetables, soil, or water contaminated by the feces of dogs^1,2^. When ingested, the eggs lose their enveloping layer in the stomach and release embryos. The embryos pass through the intestinal mucosa and reach the liver through the portal vein, where most larvae become trapped and encysted. Some larvae may pass through the capillary filter of the liver and lungs and enter the circulation to reach other organs such as the chest wall^3,4^. Combination chest wall and liver hydatid cysts are extremely rare. We present a case of hydatid cyst in chest wall associated with liver HC due to its outstanding rarity and clinical confusion with other causes of chest wall masses.

**Figure 1.**
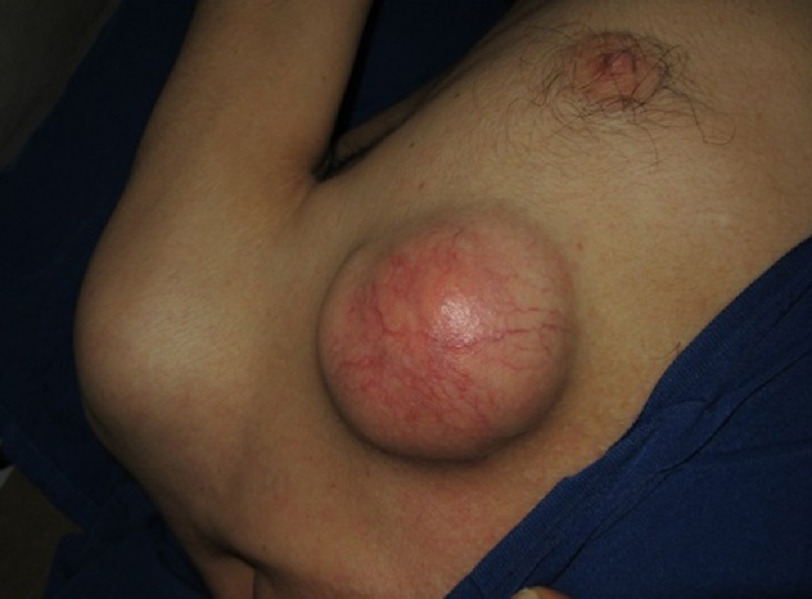
Large cystic mass forming a round structure that raised in the left anterior chest wall.

## Case presentation

A 53-year-old man presented to our center complaining of pain and swelling in the left anterior chest wall. The patient had mentioned that he had this mass for two years. He dealt with livestock for a large part of his life and had a shepherd dog. Physical examination revealed a mobile large mass in the anterior chest wall (ACW). The masses formed a round structure that raised in the left anterior chest wall (Figure 1), which were not fixed to surrounding structures. Chest wall CT scan showed no invasion to underlying structures or to ribs. Gross and microscopic examination revealed the mass as a hydatid cyst.

**Figure 2.**
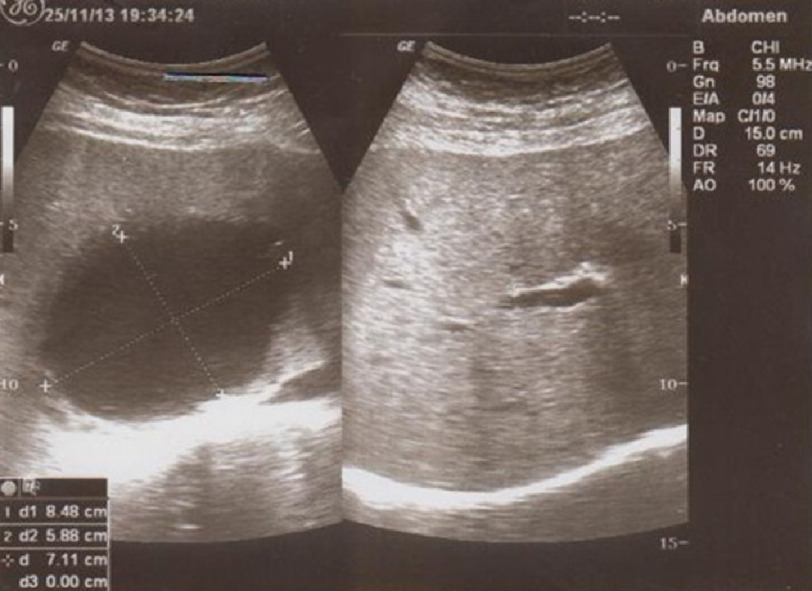
Abdominal sonography revealed a large cystic lesion in the liver.

Echinococcal indirect hemagglutination test (IHA) for *Echinococcus granulosus* was negative. It was a non-loculated hydatid cyst. Subsequently other organs were checked for HC by thoraco-abdominal computed tomography (CT) and sonography. Abdominal sonography revealed a large cystic lesion in the liver (Figure 2).

**Figure 3.**
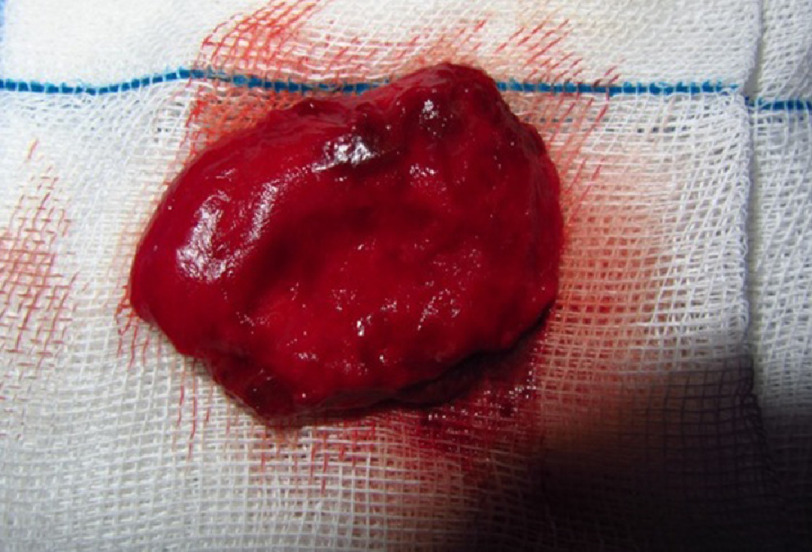
Gross cystic mass.

There was clear demarcation between the cystic lesions in chest wall and the surrounding tissue. The overlying skin, with a network of capillary vessels, gave it the appearance of a dermoid cyst. The patient was then scheduled for surgery. Under general anesthesia, the ACW was accessed using an vertical incision. The large cysts were excised completely, including the surrounding fibrous tissue (Figure 3). A drain was placed in the ACW space and surgery was completed. The patient had an uneventful postoperative course and was discharged 6 days after his operation and placed on a 4-week course of albendazole at 2 × 400 mg/d.

## Discussion

Cystic echinococcosis may affect all organs, but combined infection of the chest wall and liver is a rare event. This association may be explained by the life cycle of the parasite. The liver is the first barrier that filters the hepatic portal circulation containing 55% of HC larvae^5,6^. Most of these larvae are trapped in place in hepatic sinusoids and the structure of the cyst is created. 30% larvae that pass through the hepatic sinusoidal network of the liver will reach the lungs and may remain in this site to form primary HC.

In 15% of patients, the larvae can escape the liver and lungs blockade and reach the systemic circulation by means of the capillary system. They can then reach the anterior chest wall by six collateral arteries. In very rare cases, the larvae can also pass through the venous mesenteric lymph vessels by diffusion to enter the capillary system and systemic circulation. In pulmonary hydatidosis, the larvae can occasionally pass through the lung tissue and, by eroding to chest wall, rib and intercostal wall muscles, settle in ACW tissues^7–12^ .

Comprehension of dual HC by detection of hydatid disease with atypical localization with the liver is not easy to explain. It is difficult to describe why larvae may directly pass through the intestinal system to the systemic circulation without going to the liver or forming a liver cyst. Considering the anatomy of chest wall with abounding collateral artery, we can make some assumptions about how the hydatid cysts settle in this area.

The thoracic duct directly enters the subclavian vein and the left chest wall has a lymphatic duct that separately passes to systemic veins^13,14^. This rich lymphatic flow enables transfer of larva from the thoracic duct because in rare cases larva in the intestine can pass to the mesenteric lymph system. Related mechanisms include larval migration from the pulmonary system to the ACW or through the surrounding area to ACW.

When diagnosing ACW hydatid cysts, the primary conditions to consider are dermoid cyst, tuberculosis abscess, non-tuberculosis granuloma, lymphadenitis, parasitic diseases, hematoma, abscess, lymphocele, soft tissue sarcomas, and other malignancies causing chest wall metastasis^15,16^. Differential diagnoses can be made by a combined evaluation of physical examination, past medical history of contact with animal, CT scan, MRI, serologic tests of HC, and histopathologic examination of biopsy material^17–19^.

## Conclusion

Surgery is the most effective treatment for hydatid disease located in ACW. The main purpose of surgery is to prevent spillage of cyst to surrounding structures. Total cystectomy with surrounding adventitia, is a curative treatment for ACW hydatidosis. If dermoid cyst was not the primary diagnosis the patient should be given albendazole prophylactically 2 × 400 mg/d for at least 2 weeks. In non-complicated cases and without cyst rupture or associated other organ cyst, no postoperative medical treatment is needed.

## What we have learnt

Hydatid cysts in the anterior chest wall are an exceedingly rare complication, because the most common location of hydatid cyst is liver followed by lung and other organs. This case report demonstrates that hydatid cysts should be considered as a possible cause for palpable lesions in the chest wall, especially in endemic locations.

## Funding

This research has not received any specific grant from public, commercial, or non-profit sector agencies.

## Conflicts of interest

The authors have no conflicts of interest to declare.
